# Seroprevalence of anti-SARS-CoV-2 antibodies and risk factors among healthy blood donors in Luanda, Angola

**DOI:** 10.1186/s12879-021-06814-0

**Published:** 2021-11-02

**Authors:** Cruz S. Sebastião, Manuela Galangue, Celestina Gaston, Rui Van-Dunen, Joltim Quivinja, Emiliana Lunbungululo, Domingos Alfredo, Alberto Sozinho, Alice Teixeira, Eunice Manico, Deodete Machado, António Mateus, Zinga David, Joana Paixão, Zoraima Neto, Jocelyne Neto de Vasconcelos, Joana Morais

**Affiliations:** 1 Instituto Nacional de Investigação em Saúde (INIS), Luanda, Angola; 2 Centro de Investigação em Saúde de Angola (CISA), Caxito, Angola; 3 Instituto Superior de Ciências da Saúde (ISCISA), Universidade Agostinho Neto (UAN), Luanda, Angola; 4 Instituto Nacional de Sangue, Ministry of Health, Luanda, Angola; 5 Clínica Girassol, Ministry of Health, Luanda, Angola; 6 Faculdade de Medicina, Universidade Agostinho Neto (UAN), Luanda, Angola

**Keywords:** Seroprevalence, SARS-CoV-2, COVID-19, Blood donors, Luanda, Angola

## Abstract

**Background:**

SARS-CoV-2 emerged in China and spread throughout the world due to its rapid transmission. The exposure rate in the healthy population is unknown, mainly in resource-limited countries. Herein, we estimated the seroprevalence of anti-SARS-CoV-2 antibodies and risk factors among blood donors in Luanda, the capital city of Angola.

**Methods:**

This was a retrospective study conducted with 343 blood donors. Chi-square and logistic regression were calculated to predict the independent variable for SARS-CoV-2 infection and deemed significant when p < 0.05.

**Results:**

Seroprevalence of anti-SARS-CoV-2 was 4.7%. Positivity rates varied to age groups (3.5–14.3%), gender (0–5%), area of residence (3.1–.6%), educational level (5.1–10.2%), occupation (4.4–7.7%), and the blood donor category (2.0–5.1%). Past and recent infections were detected in 3.2% and 1.5%, respectively. Blood donors under the age of 20 years (OR: 4.58, p = 0.241) and from non-urbanized areas (OR: 1.86, p = 0.293) presented a high risk related to infection. The infection was higher in blood group A and lower in blood group O. The risk of SARS-CoV-2 infection has increased from January 2020 (OR: 0.03, p = 0.001) to August 2020 (OR: 0.57, p = 0.426).

**Conclusions:**

We provide an estimate of the exposure of healthy blood donors in Luanda. Also, we detected anti-SARS-CoV-2 in January 2020, indicating that the SARS-CoV-2 could have been imported during the first month of 2020. Further studies should be performed to assess the exposure rate in different groups from Angola.

## Introduction

In December 2019, numerous cases of pneumonia caused by the new coronavirus, the severe acute respiratory syndrome coronavirus 2 (SARS-CoV-2) emerging from Wuhan, China, were registered [1, 2]. The SARS-CoV-2 that causes coronavirus disease 2019 (COVID-19) spread rapidly throughout the world due to its rapid transmission. Until April 2021, more than 130.4 million confirmed cumulative cases and an estimated 2.9 million deaths related to COVID-19 have been reported worldwide [3]. The first SARS-CoV-2 infection case in Angola was reported in March 2020, by the Angolan Ministry of Health (MoH) [4], and until April 2021, the MoH reported more than 22 000 cases and 540 deaths related to COVID-19 [5].

Infected individuals with SARS-CoV-2 usually have mild symptoms, including fever, cough, muscle pain, anosmia, and in some cases, the infection can progress to breathing difficulties, pneumonia, or even death. However, some infected individuals have an asymptomatic SARS-CoV-2 infection, which constitutes a substantial source of transmission, as well as a potential challenge to prevent the spread of infection in the community [6–8]. The gold standard technique for detecting SARS-CoV-2 is the reverse transcription-polymerase chain reaction (RT-PCR). However, studies have shown that screen immunoglobulin M (IgM) or immunoglobulin G (IgG) antibody is useful for controlling the asymptomatic population and ensuring timely public health interventions.

Screening for infectious diseases in blood donors is essential mainly to reduce the potential risk of transmitting infectious diseases through blood transfusion [9]. Nevertheless, screening for SARS-CoV-2 in asymptomatic peoples has been little explored worldwide [10]. Currently, there is no published study assessing anti-SARS-CoV-2 antibodies in blood donors that donated before and after the identification of the first SARS-CoV-2 cases in Angola. Therefore, in the present investigation, we conducted a SARS-CoV-2 seroprevalence study among blood donors in order to identify the exposure rate in the healthy population from Luanda, the capital city and epicenter of COVID-19 in Angola.

## Materials and methods

### Study design and setting

This was a retrospective cohort study which included 343 subjects who were apparently healthy for donation at the Instituto Nacional de Sangue and Clínica Girassol, both in Luanda, the capital city of Angola, between December 2019 to February 2020 (before the first cases of SARS-CoV-2 infection were reported by the Angolan MoH) and between July to September 2020 (three months after reporting the first SARS-CoV-2 infection cases in Angola). The study was carried out at Instituto Nacional de Investigação em Saúde (INIS), located in Luanda. The INIS is a public institution of the Angolan MoH, which develops research in the most diverse areas of health and its determinants, in order to contribute to the strengthening of public health policies in Angola.

### Ethical considerations

The study was reviewed and approved by the National Ethics Committee of the Angolan MoH (approval nr. 10/2021), the direction board of the Instituto Nacional de Sangue (approval nr. 726/GDG/INS/2020), and the executive committee of the Clínica Girassol (approval nr. 0841/GEPP/PCE/2021). Moreover, anonymized data were used for analysis, and the need for individual informed consent was waived by the National Ethics Committee of the Angolan MoH for being a retrospective study.

### Sample collection and testing

Frozen plasma samples used for testing infectious disease markers at the time of donation were used for the anti-SARS-CoV-2 antibody screening. Additionally, blood donor sociodemographic characteristics were obtained by code, so that their identity would be anonymous. All methods were performed in accordance with the relevant guidelines and regulations. The samples were thawed and an estimated 5 mL aliquot of plasma was obtained from each plasma bag and stored from 2 to 8 °C, until further analysis. Sample preparation and processing were performed at the Immunoserology Laboratory of INIS. Qualitative detection of IgM/IgG antibodies against SARS-CoV-2 was performed with enzyme-linked fluorescence assays (ELFA) (bioMérieux SA, France), commercially available. This serological assay combines a two-step sandwich enzyme immunoassay method able to detect fluorescence at the end of the reaction. All processing was carried out on the mini VIDAS equipment (bioMérieux SA, France). The samples were processed following the manufacturer’s instructions. Firstly, the samples were diluted and IgG/IgM antibodies were captured by recombinant antigens contained in the strips, followed by washing for the removal of unbound components. Secondly, anti-human antibodies are labeled with alkaline phosphatase bound to IgG/IgM antibodies. Thirdly, the substrate 4-methyl-umbeliferyl phosphate was cycling in and out of the strips and the substrate was hydrolyzed to a fluorescent product (4-methyl-umbelliferone), which was subsequently measured at 450 nm. Finally, the results were calculated and all samples with a test value less than one were considered negative, while samples with a value equal to or higher than one were considered positive. Positive and negative control provided by the manufacturer were included in all reactions. The results were grouped as follows: non-infection (IgG−/IgM−), past infection (IgG+/IgM−), and recent infection (IgG−/IgM+ or IgG+/IgM+).

### Statistical analysis

The analysis was conducted in SPSS version 25 (IBM SPSS Statistics). The descriptive analysis was presented as frequencies and percentages. The normal distribution data were presented as mean and standard deviation (SD). The variables were dichotomized, analyzed with the chi-square (X^2^) test and logistic regression with a corresponding 95% confidence interval (CI) were calculated to predict the independent variable for SARS-CoV-2 infection. Additionally, an analysis of SARS-CoV-2 infection was done by the period, in order to identify the possible period of introduction and dissemination of SARS-CoV-2 infection in Luanda, the epicenter of the COVID-19 pandemic in Angola. The reported p-value is two‐tailed and was deemed statistically significant when p < 0.05.

## Results

### Seroprevalence and characteristics related to SARS-CoV-2 infection

The putative characteristics related to SARS-CoV-2 among blood donors from Luanda are summarized in Table 1. This study included a total of 343 blood donors eligible for donation between December 2019–February 2020 and between July–September 2020. Age ranged from 18 to 61 years. The mean age of the blood donors was 32 ± 9 years. Blood donors aged 20–40 years (81.3%, 279/343), men (93%, 319/343), living in non-urbanized areas (62.4%, 214/343), highly educated blood donors (76.8%, 195/343), employees (92.4%, 317/343) and family blood donors (85.7%, 294/343), were the most prevalent in the studied population. The overall seroprevalence of anti-SARS-CoV-2 antibodies was 4.7% (16/343). A total of 5/343 (1.5%) and 16/343 (4.7%) of the blood donors had IgM and IgG, respectively. About 3.2% (11/343) of the blood donors had a past infection and 1.5% (5/343) had a recent infection. High positivity rates against SARS-CoV-2 antibodies were observed in donors under the age of 20 years (14.3%), in men (5%), in non-urbanized areas (5.6%), with a low educational level (10.2%), unemployed (7.7%), and family donors (5.1%). Past infection was more frequent among blood donors under the age of 20 years (14.3%), in men (3.4%), non-urbanized areas (3.7%), in donors with a high educational level (4.6%), in unemployed donors (7.7%), and family donors (3.7%). On the other hand, recent infection was more frequent in donors over the age of 40 years (1.8%), in males (1.6%), in non-urbanized areas (1.9%), in donors with low educational levels (6.8%), employees (1.6%), and in the voluntary donors (2%). A statistically significant relationship was observed between the recent SARS-CoV-2 infection with educational level (p < 0.05), while age, gender, area of residence, occupation, and donor category showed no relationship with recent infection (p > 0.05). Besides, no relationship was observed between age, gender, area of residence, educational level, occupation, and blood donor category with anti-SARS-CoV-2 positivity and/or past infection (p > 0.05). We observed that blood donors under the age of 20 years [OR: 4.58 (95% CI: 0.36–58.4), p = 0.241] and from non-urbanized areas [OR: 1.86 (95% CI: 0.59–5.88), p = 0.293] presented a high risk for test positive against SARS-CoV-2 antibodies, compared to blood donors aged 20 years and over and from urbanized areas, respectively. On the other hand, blood donors with a high level of education [OR: 0.48 (95% CI: 0.17–1.37), p = 0.171], employed [OR: 0.55 (95% CI: 0.17–1.37), p = 0.453], and volunteers [OR: 0.39 (95% CI: 0.05–3.00), p = 0.364], presented a low risk of SARS-CoV-2 infection.

**Table 1:** Seroprevalence and putative characteristics related to SARS-CoV-2 infection among blood donors from Luanda, Angola

Independent variables	N (%)	Anti-SARS-CoV-2 positivity			Past infection			Recent infection			Univariate analysis	
		No (%)	Yes (%)	p-value*	No (%)	Yes (%)	p-value*	No (%)	Yes (%)	p-value*	OR (95% CI)	p-value
Overall	343 (100)	327 (95.3)	16 (4.7)		332 (96.8)	11 (3.2)		338 (98.5)	5 (1.5)			
*Age groups*												
< 20y	7 (2.0)	6 (85.7)	1 (14.3)	0.443	6 (85.7)	1 (14.3)	0.206	7 (100)	0 (0.0)	0.933	4.58 (0.36–58.4)	0.241
20–40y	279 (81.3)	266 (95.3)	13 (4.7)		270 (96.8)	9 (3.2)		275 (98.6)	4 (1.1)		1.34 (0.30–6.13)	0.402
> 40y	57 (16.6)	55 (96.5)	2 (3.5)		56 (98.2)	1 (1.8)		56 (98.2)	1 (1.8)		1.00	–
*Gender*												
Female	24 (7.0)	24 (100)	0 (0.0)	0.261	24 (100)	0 (0.0)	0.355	24 (100)	0 (0.0)	0.537	0 (0.0–0.0)	0.998
Male	319 (93.0)	303 (95.0)	16 (5.0)		308 (96.6)	11 (3.4)		314 (98.4)	5 (1.6)		1.00	–
*Residence area*												
Rural	214 (62.4)	202 (94.4)	12 (5.6)	0.286	206 (96.3)	8 (3.7)	0.472	210 (98.1)	4 (1.9)	0.413	1.86 (0.59–5.88)	0.293
Urban	129 (37.6)	125 (96.9)	4 (3.1)		126 (97.7)	3 (2.3)		128 (99.2)	1 (0.8)		1.00	–
*Educational level*^#^												
Low	59 (23.2)	53 (89.8)	6 (10.2)	0.163	57 (96.6)	2 (3.4)	0.685	55 (93.2)	4 (6.8)	**0**.**002**	1.00	–
High	195 (76.8)	185 (94.9)	10 (5.1)		186 (95.4)	9 (4.6)		194 (99.5)	1 (0.5)		0.48 (0.17–1.37)	0.171
*Occupation*												
Unemployed	26 (7.6)	24 (92.3)	2 (7.7)	0.446	24 (92.3)	2 (7.7)	0.177	26 (100)	0 (0.0)	0.519	1.00	–
Employed	317 (92.4)	303 (95.6)	14 (4.4)		308 (97.2)	9 (2.8)		312 (98.4)	5 (1.6)		0.55 (0.12–2.58)	0.453
*Donor category*												
Voluntary	49 (14.3)	48 (98.0)	1 (2.0)	0.347	49 (100)	0 (0.0)	0.169	48 (98.0)	1 (2.0)	0.713	0.39 (0.05–3.00)	0.364
Family	294 (85.7)	279 (94.9)	15 (5.1)		283 (96.3)	11 (3.7)		290 (98.6)	4 (1.4)		1.00	–

Past infection (IgG+/IgM−) and Recent infection (IgG−/IgM+ or IgG+/IgM+)

The bold number was statistically significant (p < 0.05)

*Chi-square test (X^2^)

^#^Missing values: 89

### Relationship between ABO/RH blood group and SARS-CoV-2 infection

The relationship between ABO/RH blood groups among blood donors from Luanda is summarized in Table 2. Blood group O (63.6%, 218/343) and a positive RH factor (97.4%, 334/343), were the most frequent. According to blood group ABO/RH, blood group ORh+ (61.8%), ARh+ (16.9%), and BRh+ (15.7%) were the most frequent. On the other hand, no blood donors had blood groups BRh− or ABRh−. The frequency of positivity for anti-SARS-CoV-2 antibodies was higher in blood group AB (20%) and less frequent in group O (3.2%). Negative donors to the RH factor showed 11.1% of anti-SARS-CoV-2 positivity, while positive donors to the RH factor accounted for 4.5%. According to the ABO/RH blood group, ARh− (33.3%) and ABRh+ (20%) blood donors showed high rates of positivity against the SARS-CoV-2 antibodies. Past infection was more frequent among blood group AB (20%), positive RH factor (3.3%), and blood group ABRh+ (20%), whereas recent infection was more frequent in blood group B (3.7%), in donors with negative RH factor (11.1%), and the blood group ARh− (33.3%). The ABO blood group was related to past infection (p = 0.023), the RH factor was related to recent infection (p = 0.014), while positivity against SARS-CoV-2 antibodies and recent infection were related with the ABO/RH blood group (p < 0.05). The risk of SARS-CoV-2 infection was much higher in group AB [OR: 7.54 (95% CI: 1.35–42.2), p = 0.022], followed by groups A [OR: 2.12 (95% CI: 0.60–7.48), p = 0.245] and B [ OR: 1.77 (95% CI: 0.44–7.10), p = 0.418], compared with group O. The risk of SARS-CoV-2 infection was also high in donors with a negative RH factor [OR: 2.66 (95% CI: 0.31–22.7), p = 0.371], in blood group ARh− [OR: 9.17 (95% CI: 0.64–132), p = 0.103], group ABRh+ [OR: 4.58 (95% CI: 0.66–31.8), p = 0.123], and group BRh+ [OR: 1.08 (95% CI: 0.21–5.59), p = 0.928]. On the other hand, a low risk of infection was observed in the blood group ORh+ [OR: 0.63 (95% CI: 0.16–2.50), p = 0.507].

**Table 2:** Relationship between ABO/RH blood group and SARS-CoV-2 infection among 343 blood donors from Luanda, Angola

Independent variables	N (%)	Anti-SARS-CoV-2 positivity			Past infection			Recent infection			Univariate analysis	
		No (%)	Yes (%)	p-value*	No (%)	Yes (%)	p-value*	No (%)	Yes (%)	p-value*	OR (95% CI)	p-value
*ABO blood group*												
A	61 (17.8)	57 (93.4)	4 (6.6)	0.075	59 (96.7)	2 (3.3)	**0**.**023**	59 (96.7)	2 (3.3)	0.174	2.12 (0.60–7.48)	0.245
B	54 (15.7)	51 (94.4)	3 (5.6)		53 (98.1)	1 (1.9)		52 (96.3)	2 (3.7)		1.77 (0.44–7.10)	0.418
AB	10 (2.9)	8 (80.0)	2 (20.0)		8 (80.0)	2 (20.0)		10 (100)	0 (0.0)		7.54 (1.35–42.2)	**0**.**022**
O	218 (63.6)	211 (96.8)	7 (3.2)		212 (97.2)	6 (2.8)		217 (99.5)	1 (0.5)		1.00	–
*RH blood group*												
RH–	9 (2.6)	8 (88.9)	1 (11.1)	0.353	9 (100)	0 (0.0)	0.580	8 (88.9)	1 (11.1)	**0**.**014**	2.66 (0.31–22.7)	0.371
RH +	334 (97.4)	319 (95.5)	15 (4.5)		323 (96.7)	11 (3.3)		330 (98.8)	4 (1.2)		1.00	–
*ABO/RH blood group*												
ARh +	58 (16.9)	55 (94.8)	3 (5.2)	**0**.**033**	56 (96.6)	2 (3.4)	0.081	57 (98.3)	1 (1.7)	** < 0**.**001**	1.00	–
ARh–	3 (0.9)	2 (66.7)	1 (33.3)		3 (100)	0 (0.0)		2 (66.7)	1 (33.3)		9.17 (0.64–132)	0.103
BRh +	54 (15.7)	51 (94.4)	3 (5.6)		53 (98.1)	1 (1.9)		52 (96.3)	2 (3.7)		1.08 (0.21–5.59)	0.928
BRh–	0 (0.0)	0 (0.0)	0 (0.0)		0 (0.0)	0 (0.0)		0 (0.0)	0 (0.0)		–	–
ABRh +	10 (2.9)	8 (80.0)	2 (20.0)		8 (80.0)	2 (20.0)		10 (100)	0 (0.0)		4.58 (0.66–31.8)	0.123
ABRh–	0 (0.0)	0 (0.0)	0 (0.0)		0 (0.0)	0 (0.0)		0 (0.0)	0 (0.0)		–	–
ORh +	212 (61.8)	205 (96.7)	7 (3.3)		206 (97.2)	6 (2.8)		211 (99.5)	1 (0.5)		0.63 (0.16–2.50)	0.507
ORh–	6 (1.7)	6 (100)	0 (0.0)		6 (100)	0 (0.0)		6 (100)	0 (0.0)		0 (0.0–0.0)	0.999

Past infection (IgG+/IgM−) and Recent infection (IgG−/IgM+ or IgG+/IgM+)

The bold numbers were statistically significant (p < 0.05)

*Chi-square test (X^2^)

### Relationship between the period of time and SARS-CoV-2 infection

The relationship between months and SARS-CoV-2 infection among blood donors from Luanda is summarized in Table 3. Blood donors carried out the donation in health units dedicated to blood collection and transfusion services during December 2019 (7.3%, 25/343), January 2020 (42.3% (145/343), February 2020 (12.2%, 42/343), July 2020 (15.2%, 52/343), August 2020 (7%, 24/343), and September 2020 (16%, 55/343). Our study detected antibodies against SARS-CoV-2 in blood donors who carried out donations in January 2020 (0.7%), July 2020 (1.69%), August 2020 (12.5%), and September 2020 (20%). The past infection was detected in August 2020 (8.3%) and September 2020 (16.4%), while the recent infection was detected in January 2020 (0.7%), July 2020 (1.9%), August 2020 (4.2%), and September 2020 (3.6%). All months included in this study before September 2020 presented a low risk of SARS-CoV-2 infection (0–12.5%). The risk of infection has increased almost 20 times, from the introduction of SARS-CoV-2 in January 2020 (OR: 0.03, p = 0.001) to August 2020 (OR: 0.08, p = 0.426).

**Table 3:** Relationship between the period of time and SARS-CoV-2 infection among 343 blood donors from Luanda, Angola

Months 2019–2020	N (%)	Anti-SARS-CoV-2 positivity			Past infection			Recent infection			Univariate analysis	
		No (%)	Yes (%)	p-value*	No (%)	Yes (%)	p-value*	No (%)	Yes (%)	p-value*	OR (95% CI)	p-value
December/2019	25 (7.3)	25 (100)	0 (0.0)	** < 0**.**001**	25 (100)	0 (0.0)	** < 0**.**001**	25 (100)	0 (0.0)	0.452	0 (0.0–0.0)	0.998
January/2020	145 (42.3)	144 (99.3)	1 (0.7)		145 (100)	0 (0.0)		144 (99.3)	1 (0.7)		0.03 (0.0–0.22)	**0**.**001**
February/2020	42 (12.2)	42 (100)	0 (0.0)		42 (100)	0 (0.0)		42 (100)	0 (0.0)		0 (0.0–0.0)	0.997
July/2020	52 (15.2)	51 (98.1)	1 (1.9)		52 (100)	0 (0.0)		51 (98.1)	1 (1.9)		0.08 (0.01–0.63)	**0**.**017**
August/2020	24 (7.0)	21 (87.5)	3 (12.5)		22 (91.7)	2 (8.3)		23 (95.8)	1 (4.2)		0.57 (0.14–2.27)	0.426
September/2020	55 (16.0)	44 (80.0)	11 (20.0)		46 (83.6)	9 (16.4)		53 (96.4)	2 (3.6)		1.00	–

Past infection (IgG+/IgM−) and Recent infection (IgG−/IgM+ or IgG+/IgM+)

The bold numbers were statistically significant (p < 0.05)

*Chi-square test (X^2^)

## Discussion

Seroprevalence studies of anti-SARS-CoV-2 antibodies can be used to estimate the cumulative number of SARS-CoV-2 infections in the population. Moreover, the antibody profile against SARS-CoV-2 in samples from healthy blood donors could represent the epidemiological situation of the population at the time of donation. To the best of our knowledge, this is the first study that assessed exposure to SARS-CoV-2 infection in a random sample of healthy blood donors who donated pre- and post-identification of the first cases of SARS-CoV-2 infection in Angola. In this survey of SARS-CoV-2 antibodies in blood donors, we found an overall seroprevalence of 4.6%, being that 1.5% positive to IgM and/or 4.7% positive to IgG. It is possible that some of these positive donors had asymptomatic SARS-CoV-2 infection, however, we cannot exclude the possibility that these donors were infectious at the time of donation. This prevalence is higher than the global prevalence of SARS-CoV-2 in Angola reported by WHO (0.02%) [5], but it is low compared to the result observed in a previous study carried out in Luanda by our research team (14.3%) [11]. The seroprevalence observed in our study is comparable to that observed in a large population-based seroepidemiological study in New York (6.9%) [12], Spain (5.0%) [13], Switzerland (4.8%) [14], and China (3.2%) [15]. Regarding blood donors, our results were high compared to the results observed in blood donors from Brazil (3.3%) [9], Italy (2.9%) [16], Denmark (1.9%) [17], Saudi Arabia (1.4%) [18], and Germany (0.91%) [19], but it was less than the results obtained in previous studies carried out with blood donors from Pakistan (21.4%) [20] and South Africa (31.8–62.5%) [21]. These differences in antibody seroprevalence might reflect a different epidemiological status between the countries. Although it is difficult to extrapolate the results of our study for the whole population from Luanda, they could suggest that SARS-CoV-2 was much more widespread than the results of the RT-PCR test showed, since these RT-PCR tests targeted symptomatic individuals, confirmed case contacts as well as individuals residing in regions with high transmission rate. This hypothesis is strongly supported, since our study showed laboratory evidence of recent SARS-CoV-2 infection (IgG+/IgM+) in January 2020 (Table 3), despite the RT-PCR tests carried out by the Angolan Ministry of Health present the first cases of SARS-CoV-2 infection only in March 2020 [4]. On the other hand, the SARS-CoV-2 infection detected in January 2020 in our study, was from an Angolan individual, resident in Luanda who has not traveled to any country with active transmission of SARS-CoV-2 before or in January 2020, as well as it had no epidemiological link with suspected individuals. Therefore, these findings could indicate that Angola already had SARS-CoV-2 infections, since January 2020, with community circulation among healthy and asymptomatic individuals before the severe cases of COVID-19 could be observed. Besides, our study showed an extremely significant increase (p < 0.001) in SARS-CoV-2 infection from 0.7% in January 2020 to 20% in September 2020 (Table 3). As expected, we observed an increase in seroprevalence over time (from January to September 2020), which can be attributed to the fact that the epidemic curve was on the rise since the introduction of the first cases of SARS-CoV-2 in Angola. Once again, it is worth mentioning that this increase in the seroprevalence of anti-SARS-CoV-2 might be the result of the rapid spread of SARS-CoV-2 infection in the community. Our results also showed that between January to September 2020, SARS-CoV-2 infection evolved mainly from non-urbanized regions (5.6%) to urbanized regions (3.1%). This hypothesis is also supported by the fact that the rate of recent infection in non-urbanized regions (1.9%) is higher compared to the rate observed in urbanized regions (0.8%), although the difference is not significant (p = 0.413). Also, our study showed that the risk of SARS-CoV-2 infection was 1.86 times in non-urbanized regions when compared to urbanized regions (Table 1). Weak basic sanitation and low socioeconomic status could help explain the increase in SARS-CoV-2 infections in non-urbanized regions of Luanda since in these regions, the population might have economic difficulties which prevent the acquisition of protective materials or the fulfillment of social isolation to prevent the spread of SARS-CoV-2 infection.

All blood donors who tested positive for SARS-CoV-2 in our study were male (Table 1). Despite no observed significance (p > 0.05), other studies have also observed a higher rate of SARS-CoV-2 infection in male blood donors compared to female blood donors [22]. One of the possible explanations is the fact that the majority of blood donors in Luanda are male (93%) compared to female (7%). A higher frequency of male blood donors has also been observed in Pakistan [20]. In Angola, the population up to 14 years old represents 47%, from 15 to 64 years old it represents 50% and equal to or above 65 years old represents only 2% of the population [23]. These data are similar to those observed in Kenya, where also only 3% of the population constitutes the age group above or equal to 65 years old, but it differs from the findings of a study carried out in Italy, where more than 20% of the population he was 65 years of age or older [24]. The fact that the majority of the Angolan population is under the age of 65 years could help explain the increase in asymptomatic cases as well as the reduction of severe cases or deaths related to COVID-19 in the Angolan community [5]. Consequently, the demographic age pyramid in Angola results in a group of individuals at a younger age vulnerable to SARS-CoV-2 infection. For example, in our previous study, we showed that the infection rate as well as the risk of infection in the general population of Luanda, increases with increasing age [11], however, in this study, we showed that the risk of exposure reduces with increasing age (14.3% for blood donors under 20 years old and 3.5% for blood donors over 40 years old). Furthermore, blood donors under the age of 20 years had a higher risk of SARS-CoV-2 infection (OR: 4.58, p = 0.241), compared to blood donors over the age of 20 years (Table 1). Our study already expected greater seroprevalence in younger donors, since in addition to being more likely to get around, make up the core of the workforce, which increases the exposure rate to SARS-CoV-2 despite the social distance imposed by health authorities. A study carried out with blood donors from Brazil [9] and Kenya [24] also observed a reduction in the SARS-CoV-2 infection rate with increasing age, while contrary results in which adult individuals were more exposed were observed in Denmark [17], Saudi Arabia [18], and Iran [25].

We already expected higher seroprevalence and risk of SARS-CoV-2 infection among less-educated blood donors, in non-urbanized regions, unemployed, and family blood donors of patients who might receive the donated blood components (Table 1). Due to sociodemographic characteristics, these groups of individuals belong to a low socioeconomic stratum, lived in dwellings without a basic sanitation system, and have high difficulty in fulfilling social distance or adhering to basic hygiene measures to control SARS-CoV-2 dissemination. Unlike other studies in which seroprevalence estimates did not vary by occupation [25], our results indicate a higher risk of infection in unemployed blood donors compared to employed blood donors, which is different from the results obtained in South Africa, where high seroprevalence of SARS-CoV-2 was observed in employed [26]. On the other hand, similar to our study, where infection in highly educated blood donors was lower (OR: 0.48, p = 0.171), a study carried out in Brazil reported a high risk of infection in blood donors with low education (OR: 1.72, p = 0.011) [9]. Furthermore, we observed a higher risk in non-urbanized regions (OR: 1.86, p = 0.293) and in Brazil, they observed less risk in urbanized regions (OR: 0.86, p = 0.464) [9], indicating on the one hand that individuals with high socioeconomic status tend to comply more with the measures imposed by health facilities to prevent the dissemination of the SARS-CoV-2 infection and on the other hand that the infection might be easily controlled in the population with a high socioeconomic level. However, results contrary to those observed in our study and Brazil were observed in Canada, where blood donors from underserved areas did not have significantly higher rates of SARS-CoV-2 infection compared to blood donors from more affluent neighborhoods [22].

Recent studies have shown the existence of an association between ABO blood groups with SARS-CoV-2 and another coronavirus [27, 28]. We observed a significant difference between ABO/RH blood groups with SARS-CoV-2 infection (p = 0.033) (Table 2). Similar to that reported in previous studies [20], we found that blood group A increases the risk of SARS-CoV-2 infection, whereas blood group O decreases the risk of SARS-CoV-2 infection (Table 2). Within-group A, we observed an increase from 5.2% in ARh+ to 33.3% in ARh−, while all non-A groups had a decrease from the Rh+ to the Rh−, for example, group B decreased from 5.6% (BRh+) to 0% (BRh−), blood group AB decreased from 20% (ABRh+) to 0% (ABRh−), and blood group O decreased from 3.3% (ORh+) to 0% (ORh−). The reason for the high risk of SARS-CoV-2 infection in blood group A compared to all non-A groups remains unknown and needs further investigation, although some studies showed that the protection mechanism of circulating anti-A antibodies inhibits the interaction between SARS-CoV-2 and the ACE2 receptor [27–30]. Consequently, blood group A individuals might need reinforcing protection to reduce the chance of getting the SARS-CoV-2 infection or in case of infection, reinforcement of clinical surveillance and aggressive treatment to avoid the severity of the infection. However, further studies on the relationship between ABO/RH blood groups, the COVID-19 severity, and clinical outcome should be carried out to support Angolan health authorities in defining strategies able to reduce the COVID-19 severity according to ABO/RH blood group.

It is worth mentioning that our study is accompanied by limitations especially regarding the representativeness of the population. Therefore, patterns of SARS-CoV-2 susceptibility among blood donors might differ from the general population. In this study, young people and adults aged ranging between 18 and 61 years, are overrepresented compared to the underrepresented group which included children, the elderly, and individuals with some limitation or infectious disease. Furthermore, were not performed viral load, antibody cross-reactivity, and screening with other tests with high sensitivity and/or specificity in the reactive and non-reactive samples of this study as well as we did not perform antibody titer quantification due to resource limitations. Based on these limitations, our results might suggest the possibility of numerous unreported cases of SARS-CoV-2 infection during the period in which these blood donors donated blood in Luanda. Moreover, since the products derived from the transfusions of these blood donors are indicated for immunodeficient patients and other therapeutic and/or prophylactic approaches, we suggest that the Angolan Ministry of Health should consider the possibility to screen IgG and IgM antibodies in all blood donation candidates before performing blood donation to prevent transmission of the virus through blood transfusion in Angola. Despite this, our findings encourage further studies with blood donors and/or other groups from different regions of Angola.

## Conclusions

The data obtained from the present study provide an estimate of the exposure to SARS-CoV-2 infection among healthy blood donors in Luanda, the capital city of Angola. Moreover, our results indicate that (i) SARS-CoV-2 might have been introduced in January 2020, (ii) SARS-CoV-2 seroprevalence has increased over a period of time, and (iii) the exposure rate might be higher than that reported based on the molecular assay. Continuous screening for anti-SARS-CoV-2 in blood donors and/or other groups could be an important tool to monitor the extent of the SARS-CoV-2 infection and support authorities in decision-making for the management of the COVID-19 pandemic in Angola.

## Data Availability

All relevant data are within the paper.

## References

[1] Zhu N, Zhang D, Wang W, Li X, Yang B, Song J (2020). A Novel Coronavirus from Patients with Pneumonia in China, 2019. N Engl J Med.

[2] Li Q, Guan X, Wu P, Wang X, Zhou L, Tong Y (2020). Early Transmission Dynamics in Wuhan, China, of Novel Coronavirus–Infected Pneumonia. N Engl J Med.

[3] World Health Organization. COVID-19 Weekly Epidemiological Update. WHO. 2020; November:1;4. https://www.who.int/docs/default-source/coronaviruse/situation-reports/20201012-weekly-epi-update-9.pdf.

[4] Ministério da Saúde de Angola. Pandemia da COVID-19 em Angola. Boletin informativo 269: 16 de outubro de 2020. 2020;:58–9.

[5] World Health Organization. Weekly epidemiological update on COVID-19 - 6 April 2021. World Heal Organ. 2021; December:1–3. https://www.who.int/docs/default-source/coronaviruse/situation-reports/weekly_epidemiological_update_22.pdf.

[6] Jin ZJ, Dong X, Yuan CY, Dong YY, Bin YY, Qin YY (2020). Clinical characteristics of 140 patients infected with SARS-CoV-2 in Wuhan, China. Allergy Eur J Allergy Clin Immunol..

[7] Zhou F, Yu T, Du R, Fan G, Liu Y, Liu Z (2020). Clinical course and risk factors for mortality of adult inpatients with COVID-19 in Wuhan, China: a retrospective cohort study. Lancet.

[8] Guan WJ, Ni ZY, Hu Y, Liang WH, Ou CQ, He JX (2020). Clinical Characteristics of Coronavirus Disease 2019 in China. N Engl J Med.

[9] Filho LA, Szwarcwald CL, Mateos S, dede OGLeon ACMP, de Andrade Medronho R, Veloso VG (2020). Seroprevalence of anti-SARS-CoV-2 among blood donors in Rio de Janeiro, Brazil. Rev Saude Publica.

[10] Chang L, Yan Y, Wang L (2020). Coronavirus Disease 2019: Coronaviruses and blood safety. Transfus Med Rev..

[11] Sebastião CS, Neto Z, Martinez P, Jandondo D, Antonio J, Galangue M (2021). Sociodemographic characteristics and risk factors related to SARS-CoV-2 infection in Luanda, Angola. PLoS One.

[12] Havers FP, Reed C, Lim T, Montgomery JM, Klena JD, Hall AJ (2020). Seroprevalence of Antibodies to SARS-CoV-2 in 10 Sites in the United States, March 23-May 12, 2020. JAMA Intern Med.

[13] Pollán M, Pérez-Gómez B, Pastor-Barriuso R, Oteo J, Hernán MA, Pérez-Olmeda M (2020). Prevalence of SARS-CoV-2 in Spain (ENE-COVID): a nationwide, population-based seroepidemiological study. Lancet.

[14] Stringhini S, Wisniak A, Piumatti G, Azman AS, Lauer SA, Baysson H (2020). Seroprevalence of anti-SARS-CoV-2 IgG antibodies in Geneva, Switzerland (SEROCoV-POP): a population-based study. Lancet.

[15] Xu X, Sun J, Nie S, Li H, Kong Y, Liang M (2020). Seroprevalence of immunoglobulin M and G antibodies against SARS-CoV-2 in China. Nat Med.

[16] Valenti L, Bergna A, Pelusi S, Facciotti F, Lai A, Tarkowski M (2021). SARS-CoV-2 seroprevalence trends in healthy blood donors during the COVID-19 outbreak in Milan. Blood Transfus.

[17] Erikstrup C, Hother CE, Pedersen OBV, Mølbak K, Skov RL, Holm DK (2021). Estimation of SARS-CoV-2 Infection Fatality Rate by Real-time Antibody Screening of Blood Donors. Clin Infect Dis.

[18] Banjar A, Al-Tawfiq JA, Alruwaily A, Alserehi H, Al-Qunaibet A, Alaswad R (2021). Seroprevalence of antibodies to SARS-CoV-2 among blood donors in the early months of the pandemic in Saudi Arabia. Int J Infect Dis.

[19] Fischer B, Knabbe C, Vollmer T (2020). SARS-CoV-2 IgG seroprevalence in blood donors located in three different federal states, Germany, March to June 2020. Eurosurveillance.

[20] Younas A, Waheed S, Khawaja S, Imam M, Borhany M, Shamsi T. Seroprevalence of SARS-CoV-2 antibodies among healthy blood donors in Karachi, Pakistan. Transfus Apher Sci. 2020;59(6):102923. 10.1016/j.transci.2020.102923. 10.1016/j.transci.2020.102923 PMC7444608 32868226

[21] Sykes W, Mhlanga L, Swanevelder R, Glatt TN, Grebe E, Coleman C (2021). Prevalence of anti-SARS-CoV-2 antibodies among blood donors in Northern Cape, KwaZulu-Natal, Eastern Cape, and Free State provinces of South Africa in January 2021. Res Sq.

[22] Saeed S, Drews SJ, Pambrun C, Yi QL, Osmond L, O’Brien SF (2021). SARS-CoV-2 seroprevalence among blood donors after the first COVID-19 wave in Canada. Transfusion.

[23] National Institute of Statistics. Resultados definitivos do recenseamento geral da população e da habitação de Angola - 2014. 2016. http://www.effaangola.org/AngolaCensus2014_ResultadosDefinitivos_Mar2016.pdf.

[24] Uyoga S, Adetifa IMO, Karanja HK, Nyagwange J, Tuju J, Wanjiku P (2020). Seroprevalence of anti-SARS-CoV-2 IgG antibodies in Kenyan blood donors. medRxiv.

[25] Poustchi H, Darvishian M, Mohammadi Z, Shayanrad A, Delavari A, Bahadorimonfared A (2021). SARS-CoV-2 antibody seroprevalence in the general population and high-risk occupational groups across 18 cities in Iran: a population-based cross-sectional study. Lancet Infect Dis.

[26] Shaw JA, Meiring M, Cummins T, Chegou NN, Claassen C, Du Plessis N (2021). Higher SARS-CoV-2 seroprevalence in workers with lower socioeconomic status in Cape Town, South Africa. PLoS One.

[27] Zhao J, Yang Y, Huang H, Li D, Gu D, Lu X, et al. Relationship Between the ABO Blood Group and the Coronavirus Disease 2019 (COVID-19) Susceptibility. Clin Infect Dis. 2020;:1–35. doi:10.1093/cid/ciaa1150. 10.1093/cid/ciaa1150 PMC7454371 32750119

[28] Jokela M, Virtanen M, David Batty G, Kivimaki M (2005). ABO Blood Group and Susceptibility to Severe Acute Respiratory Syndrome. JAMA.

[29] Li J, Wang X, Chen J, Cai Y, Deng A, Yang M (2020). Association between ABO blood groups and risk of SARS-CoV-2 pneumonia. Br J Haematol.

[30] Huang C, Wang Y, Li X, Ren L, Zhao J, Hu Y, Zhang L, Fan G, Xu J, Gu X, Cheng Z, Yu T, Xia J, Wei Y, Wu W, Xie X, Yin W, Li H, Liu M, Xiao Y, Gao H, Guo L, Xie J, Wang G, Jiang R, Gao Z, Jin Q, Wang J, Cao B. Clinical features of patients infected with 2019 novel coronavirus in Wuhan, China. Lancet. 2020;395(10223):497–506. 10.1016/S0140-6736(20)30183-5. 10.1016/S0140-6736(20)30183-5 PMC7159299 31986264

